# Venous oxygen saturation estimation from multiple T2 maps with varying inter-echo spacing

**DOI:** 10.1186/1532-429X-18-S1-W29

**Published:** 2016-01-27

**Authors:** Juliet Varghese, Rizwan Ahmad, Ning Jin, Lee C Potter, Orlando P Simonetti

**Affiliations:** 1 Department of Biomedical Engineering, The Ohio State University, Columbus, OH USA; 2 Dorothy M. Davis Heart and Lung Research Institute, The Ohio State University Wexner Medical Center, Columbus, OH USA; 3 Department of Electrical and Computer Engineering, The Ohio State University, Columbus, OH USA; 4 Siemens Healthcare, Columbus, OH USA; 5 Division of Cardiovascular Medicine, Department of Internal Medicine, The Ohio State University Wexner Medical Center, Columbus, OH USA; 6 Department of Radiology, The Ohio State University Wexner Medical Center, Columbus, OH USA

## Background

Dependence of blood T2 on O2 saturation has led to non-invasive MRI-based techniques for determining venous O2 saturation (SvO_2_) [1-3]. However, applying a general calibration factor derived from in vitro experiments can lead to inaccurate and largely varying SvO_2_ estimates in the target population. We aim to show that based on the Luz-Meiboom relation 1/T2 = 1/T2o + Hct(1-Hct) τ_ex_[(1-%SO_2_/100)αω_0_]^2^(1-2*τ_ex_/τ_180_ tanh(τ_180_/2*τ_ex_))[4], individual SvO_2_ can be determined from multiple T2 maps, each acquired at a specific inter-echo spacing (τ_180_).

## Methods

Three T2 prepared SSFP quantitative T2 maps (τ_180_=10, 12 and 15 ms, TR >3000 ms, FA = 40^0^, 2.8 × 2.8 × 10 mm^3^, 2NEX, free breathing) were acquired in seven volunteers (age: 32.6 ± 12.2 yrs) on a 3T MRI system (Tim Trio, Siemens Healthcare). T2 preparation involved an MLEV refocusing pulse train with 2, 4, 8 and 12 composite pulses. Venous and arterial blood T2 were measured in each map in an ROI in the ventricles. For each subject, the multiple τ_180_ measurements were processed jointly to estimate SvO_2_ along with other nuisance parameters (T2o and τ_ex_) via constrained nonlinear least squares fitting in Matlab (The Mathworks Inc, Natick, MA, USA). The values of hematocrit (Hct), arterial O2 saturation (SaO_2_), and α were fixed at 41%, 97%, and 0.4 ppm respectively.

## Results

**Figure 1:**
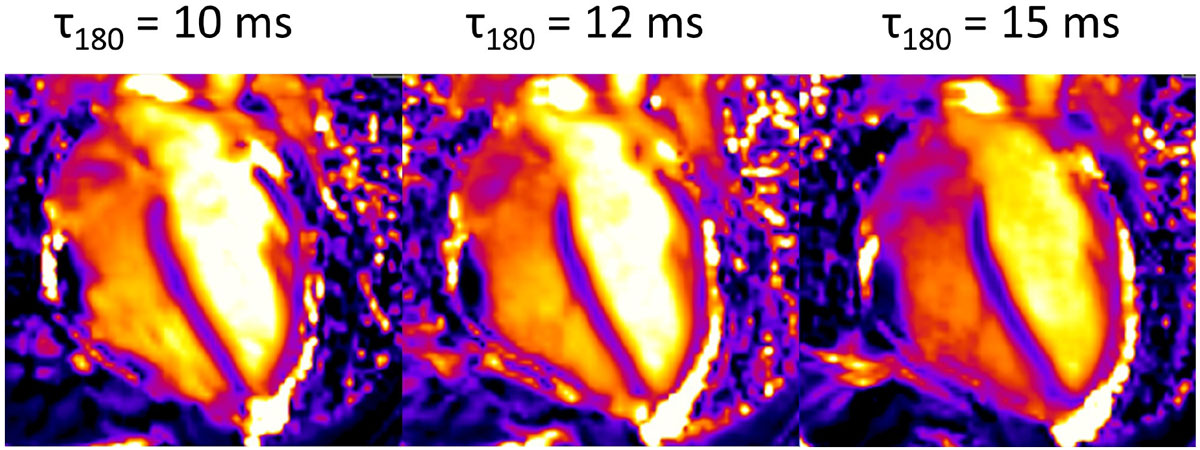
**T2 prepared SSFP quantitative T2 maps (four-chamber view) acquired in a volunteer at different τ**_**180**_ **(10, 12 and 15 ms)**.

Figure 1 shows T2 maps acquired in a volunteer. Table 1 shows mean ± SD of venous and arterial T2 and estimated parameters. Venous and arterial blood T2 agree with previously reported values at 3T [2,3]. Estimated SvO_2_ for all volunteers falls within the normal physiological range (60-80%).

**Table 1:** Table 1

τ180 (ms)	TET2p (ms)	Venous T2 (ms)	Arterial T2 (ms)	Estimated Parameters
10	0,20,40,80,120	125.7 ± 11.7	169.4 ± 12.8	SvO2: 72.6 ± 5.1% T2o: 168.6 ± 12.8 ms τex: 5.4 ± 3.5 ms
12	0,24,48,96,144	122.2 ± 9.8	176.4 ± 22.6	
15	0,30,60,120	110.6 ± 13.2	161.2 ± 16.7	

The T2 preparation times (TET2p) at which T2 maps were acquired for a specific τ180, the mean ± SD of venous and arterial T2 values measured in an ROI in the right and left ventricles in all volunteers, and mean ± SD of the parameters estimated from the maps.

## Conclusions

The measured T2 of blood is dependent on Hct, O2 saturation, and τ_180_. We have shown that if these parameters are known, SvO_2_ can be non-invasively determined from arterial and venous blood T2 maps acquired at multiple τ_180_. This provides in-vivo, patient-specific calibration, and may reduce the uncertainty and error in SvO2 estimation from applying a general calibration factor to the entire patient population. Although in this preliminary study we have assumed a fixed Hct and SaO_2_ for all subjects, greater accuracy may be achieved by measuring individual Hct and SaO_2_ from a blood sample and a pulse oximeter respectively. Nevertheless, our results show that the proposed approach is feasible, giving reasonable SvO_2_ estimates. Future studies will involve validation of the single assumed parameter, α, in the T2-SO_2_ model, optimization of the set of τ_180_ times, and further evaluation of the accuracy and precision of T2 mapping in determining blood O2 saturation. This would lead to the development of rapid, accurate, non-invasive, in-vivo quantification of SvO_2_ from T2 maps, which would be highly beneficial for heart failure and congenital heart disease patients.
